# Transforming Properties of Beta-3 Human Papillomavirus E6 and E7 Proteins

**DOI:** 10.1128/mSphere.00398-20

**Published:** 2020-07-15

**Authors:** Lucia Minoni, Maria Carmen Romero-Medina, Assunta Venuti, Cécilia Sirand, Alexis Robitaille, Gennaro Altamura, Florence Le Calvez-Kelm, Daniele Viarisio, Katia Zanier, Martin Müller, Rosita Accardi, Massimo Tommasino

**Affiliations:** a International Agency for Research on Cancer (IARC), World Health Organization, Lyon, France; b Deutsches Krebsforschungszentrum (DKFZ), Heidelberg, Germany; c Department of Veterinary Medicine and Animal Productions, University of Naples Federico II, Naples, Italy; d Equipe labellisée Ligue, Biotechnologie et signalisation cellulaire UMR 7242, Ecole Supérieure de Biotechnologie de Strasbourg, Illkirch, France; University of Pittsburgh

**Keywords:** beta-3 and mucosal HPV types, p53 and pRb regulated pathways, keratinocyte immortalization

## Abstract

The beta human papillomaviruses (HPVs) are subdivided into 5 species (beta-1 to beta-5), and they were first identified in the skin. However, the beta-3 species appears to be more highly represented in the mucosal epithelia than in the skin. Functional studies have also highlighted that beta-3 HPV49 shares some functional similarities with mucosal high-risk (HR) HPV16. Here, we describe the characterization of the *in vitro* transforming properties of the entire beta-3 species, which includes three additional HPV types: HPV75, HPV76, and HPV115. HPV49, HPV75, and HPV76 E6 and E7 (E6/E7), but not HPV115 E6 and E7, efficiently inactivate the p53 and pRb pathways and immortalize or extend the life span of human foreskin keratinocytes (HFKs). As observed for HR HPV16, cell cycle deregulation mediated by beta-3 HPV E6/E7 expression leads to p16^INK4a^ accumulation, whereas no p16^INK4a^ was detected in beta-2 HPV38 E6/E7 HFKs. As shown for HPV49 E6, HPV75 and HPV76 E6s degrade p53 by an E6AP/proteasome-mediated mechanism. Comparative analysis of cellular gene expression patterns of HFKs containing E6 and E7 from HR HPV16, beta-3 HPV types, and beta-2 HPV38 further highlights the functional similarities of HR HPV16 and beta-3 HPV49, HPV75, and HPV76. The expression profiles of these four HPV HFKs show some similarities and diverge substantially from those of beta-3 HPV115 E6/E7 and beta-2 HPV38 E6/E7 HFKs. In summary, our data show that beta-3 HPV types share some mechanisms with HR HPV types and pave the way for additional studies aiming to evaluate their potential role in human pathologies.

**IMPORTANCE** Human papillomaviruses are currently classified in different genera. Mucosal HPVs belonging to the alpha genus have been clearly associated with carcinogenesis of the mucosal epithelium at different sites. Beta HPV types have been classified as cutaneous. Although findings indicate that some beta HPVs from species 1 and 2 play a role, together with UV irradiation, in skin cancer, very little is known about the transforming properties of most of the beta HPVs. This report shows the transforming activity of E6 and E7 from beta-3 HPV types. Moreover, it highlights that beta-3 HPVs share some biological properties more extensively with mucosal high-risk HPV16 than with beta-2 HPV38. This report provides new paradigms for a better understanding of the biology of the different HPV types and their possible association with lesions at mucosal and/or cutaneous epithelia.

## Abstract (precis)

Human papillomaviruses are currently classified in different genera. Mucosal HPVs belonging to the alpha genus have been clearly associated with carcinogenesis of the mucosal epithelium at different sites. Beta HPV types have been classified as cutaneous. Although findings indicate that some beta HPVs from species 1 and 2 play a role, together with UV irradiation, in skin cancer, very little is known about the transforming properties of most of the beta HPVs. This report shows the transforming activity of E6 and E7 from beta-3 HPV types. Moreover, it highlights that beta-3 HPVs share some biological properties more extensively with mucosal high-risk HPV16 than with beta-2 HPV38. This report provides new paradigms for a better understanding of the biology of the different HPV types and their possible association with lesions at mucosal and/or cutaneous epithelia.

## INTRODUCTION

To date, more than 200 human papillomavirus (HPV) types have been isolated from different anatomical sites and fully sequenced. On the basis of the sequences of the major capsid protein L1, HPVs are classified into genera, species, and types (1, 2). The alpha, beta, and gamma genera are the largest subgroups and include most of the HPV types isolated so far. Genus alpha comprises the mucosal high-risk (HR) HPV types that have been associated with cervical cancer and with a subset of other genital cancers as well as with a subset of oropharyngeal cancer (3). Beta HPV types are considered to have a cutaneous tropism and, together with UV radiation, are suspected to promote skin squamous cell carcinoma (sSCC) (4). Approximately 50 beta HPV types have been fully characterized and are subgrouped into five species (beta-1 to beta-5). The first beta HPV types identified, HPV5 and HPV8, were isolated from patients suffering from a rare autosomal recessive genetic disorder called epidermodysplasia verruciformis (EV) (4–6). Moreover, many epidemiological findings support the association of beta HPVs and sSCC also in the general population. Specifically, non-EV sSCC patients are more frequently positive for viral markers (DNA and/or antibodies) than are healthy individuals (6–14).

Recent findings indicate that beta HPV types can also infect other anatomical sites in addition to the skin, such as the oral mucosal epithelium, eyebrow hairs, and penile and external genital lesions (15–20). Although no findings support the direct involvement of beta HPV types in pathological conditions at any of the anatomical sites mentioned above, a prospective study showed that DNA positivity for some beta HPV types in the oral cavity was associated with a risk of head and neck cancer (21). It has been also reported that the beta-3 species, which include only four HPV types (49, 75, 76, and 115), are more prevalent in some mucosal epithelia than in the skin (17, 22). Although only a limited number of beta HPV types have been subjected to mechanistic studies (4), findings indicate that E6 and E7 (E6/E7) from beta-3 HPV49 and HR HPV16 share some functional similarities, including efficient immortalization of primary human foreskin keratinocytes (HFKs) (23). In addition, HPV49 E6, like HPV16 E6, binds the ubiquitin E3 ligase enzyme E6AP, promoting p53 degradation via the proteasome pathway. In contrast, other beta HPV types have developed completely different mechanisms of altering p53 functions (4). For instance, beta-2 HPV38 E7 induces accumulation of ΔNp73α, an antagonist of p53 transcriptional functions (24, 25).

Transgenic (Tg) mouse models expressing E6 and E7 in the basal layer of the epithelium under the control of the keratin 14 (K14) promoter provide further evidence for the functional similarities between HPV types 16 and 49. K14 beta-3 HPV49 or alpha HPV16 E6-Tg and E7-Tg (E6/E7-Tg) animals were found to be highly susceptible to upper digestive tract carcinogenesis upon initiation with 4-nitroquinoline 1-oxide (4NQO), whereas K14 beta-2 HPV38 E6/E7-Tg mice were not affected much by 4NQO treatment (26, 27).

Together, these findings indicate that within the genus beta, the beta-3 species may represent a subgroup that shares some properties with alpha HR HPV types. However, except for HPV49, very little is known about the biology of the other beta-3 HPV types.

In this work, we performed a comparative study on the biological properties of all four beta-3 HPV types and confirmed that this species is biologically related to mucosal HR HPV types.

## RESULTS

### E6 and E7 from beta HPV49 and from HPV76 efficiently immortalize HFKs.

**FIG 1:**
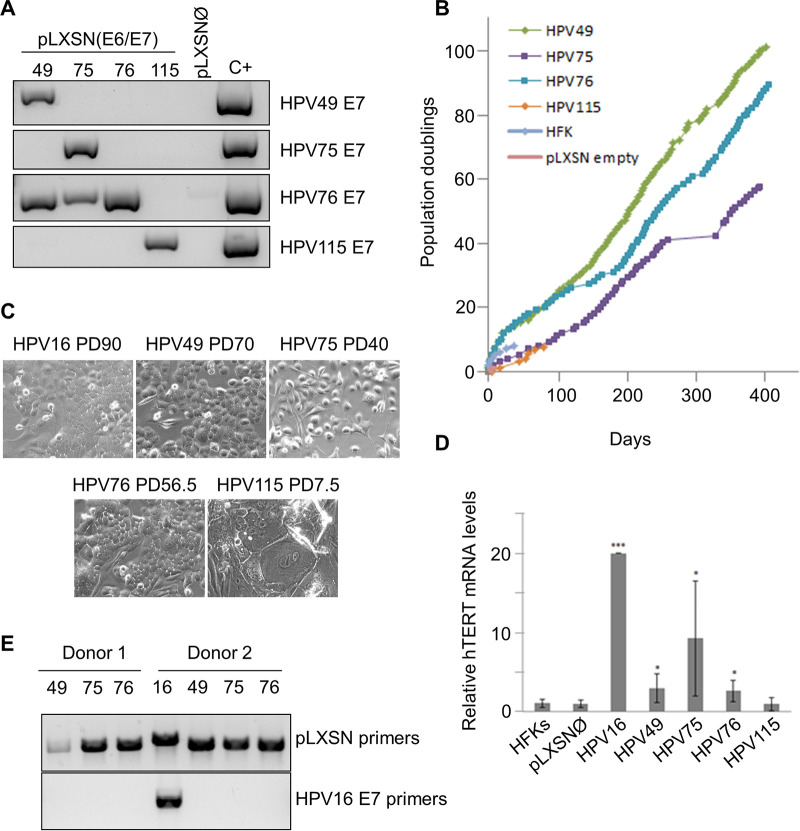
Stable expression of E6/E7 of HPV49 and HPV76 immortalizes primary HFKs. (A) Total RNA was extracted from HFKs transduced with the indicated retroviruses and subjected to retrotranscription. PCR was performed using different cDNAs as templates with HPV E7-specific primers. (B) Growth curve of HFKs (donor 1) expressing E6/E7 from the indicated HPVs. (C) Morphology of HFKs transduced with the indicated recombinant retroviruses at the indicated PDs. The same magnification (×20) was used for all the microphotographs. (D) Total RNA extracted from cells at the same passage was retrotranscribed and used as a template for RT–real-time PCR analysis of hTERT gene expression, normalized to GAPDH. Each result shown in the histogram represents the mean of results from three independent experiments performed in two donors (*, *P* < 0.05; ***, *P* < 0.005). (E) Total DNA was extracted from HFKs transduced with the indicated retroviruses and subjected to PCR using specific pLXSN primers recognizing regions immediately before and after the viral genes (top panel). Portions of these PCR products were used for a second PCR performed with HPV16 E7 primers (bottom panel).

We first compared the abilities of E6 and E7 from all beta-3 HPV types to immortalize primary HFKs. HFKs were transduced with empty retroviral vector (pLXSNØ) or with vector carrying E6/E7 genes from all the beta-3 HPVs. Retrotransduced HFKs were analyzed for the expression levels of of the E6/E7 polycistronic transcript by reverse transcriptase-PCR (RT-PCR) (Fig. 1A). Due to genome similarities within beta-3 species, the HPV76 PCR primers also amplify the retrotranscribed HPV49 and HPV75 mRNA, whereas the primers for HPV49, HPV75, and HPV115 showed high specificity (Fig. 1A). Expression of HPV49, HPV75, and HPV76 E6/E7 induced continuous growth of HFKs, which had reached 60 to 100 population doublings (PDs) to the date of assay (Fig. 1B). In contrast, HPV115 E6/E7-transduced HFKs were able to proliferate for only a few PDs and died at approximately the same time as the HFKs transduced with empty pLXSN (Fig. 1B). Transduction with the different recombinant retroviruses was repeated with HFKs from two additional donors (Table 1). Long-term culture of the different HFKs revealed that, similarly to what we previously showed for HPV49, E6 and E7 proteins from HPV76 and HPV75 were able to increase the life span of HFKs. However, to the date of assay, only HPV49 and HPV76 E6/E7-expressing HFKs from donor 1 had reached more than 100 PDs, which was an indication of immortalization (Fig. 1B; see also Table 1). HPV75 E6/E7 HFKs from the same donor (donor 1) appeared to proliferate at lower levels than HPV49 and HPV76 E6/E7 HFKs and reached approximately 60 PDs (Fig. 1B; see also Table 1). However, we observed no differences in the proliferation rates of HPV49, HPV75, and HPV76 E6/E7 HFKs from donor 2 (Table 1), suggesting that, at least for HPV75 E6/E7, the genetic makeup of the host may influence the efficiency of HPV75 in promoting cellular proliferation.

**TABLE 1:** E6 and E7 from beta-3 HPV differentially affect cell growth ability when retrotransduced in primary keratinocytes^*a*^

Retrovirus	Outcome to date		
	Donor 1	Donor 2	Donor 3
pLXSNØ	Dead at 1 PD	Dead at 1 PD	Dead at 1 PD
HPV49	108 PD	54 PD	5 PD
HPV75	63.5 PD	50.5 PD	ND
HPV76	101 PD	49.5 PD	6 PD
HPV115	Dead at 7.5 PD	Dead at 1 PD	Dead at 1 PD

a Data represent the number of population doublings (PDs) reached to date by the indicated keratinocytes (from 3 different donors) stably expressing E6 and E7 of beta-3 HPVs or carrying the empty retroviral vector (pLXSNØ). ND, not done.

In line with the low proliferation rate, HPV115 E6/E7 HFKs displayed features of arrested and senescent cells, such as irregular shapes, intercellular bridges, and large and multinucleated cells (Fig. 1C). In contrast, the HFKs expressing the E6/E7 of the other beta-3 HPV types showed the typical morphology of highly proliferative cells, characterized by small size, high brightness, and regular shape (Fig. 1C). Activation of human telomerase reverse transcriptase (hTERT) is a key step in HPV-induced HFK immortalization (4). Quantitative RT-PCR revealed that HPV49, HPV75, and HPV76 showed increased hTERT expression, although at levels lower than those seen with HPV16 (Fig. 1D). In contrast, HPV115 E6/E7 HFKs express very low hTERT mRNA levels similar to those seen with untransduced HFKs or with HFKs transduced with empty retrovirus (Fig. 1D).

Finally, to exclude the remote possibility that immortalized HPV16 E6/E7 HFKs had contaminated the beta-3 HPV HFK cultures, leading to misinterpretation of the results, we extracted the DNA from the different HFK cultures and amplified the E6/E7 region using primers designed to correspond to pLXSN regions located immediately before and after the viral genes (Fig. 1E, top panel). Direct DNA sequencing of the PCR products confirmed that no cross contamination had occurred among the different HFK cultures (data not shown). In addition, the same PCR products were used as templates for an additional PCR with specific HPV16 E7 primers. Only one PCR product was observed in the template corresponding to HPV16 E6/E7 HFKs (Fig. 1E, bottom panel).

Together, these data highlight some differences in the *in vitro* transforming properties of the beta-3 HPV types.

### The pRb pathway and cell cycle control are altered in beta-3 HPV E6/E7 HFKs.

**FIG 2:**
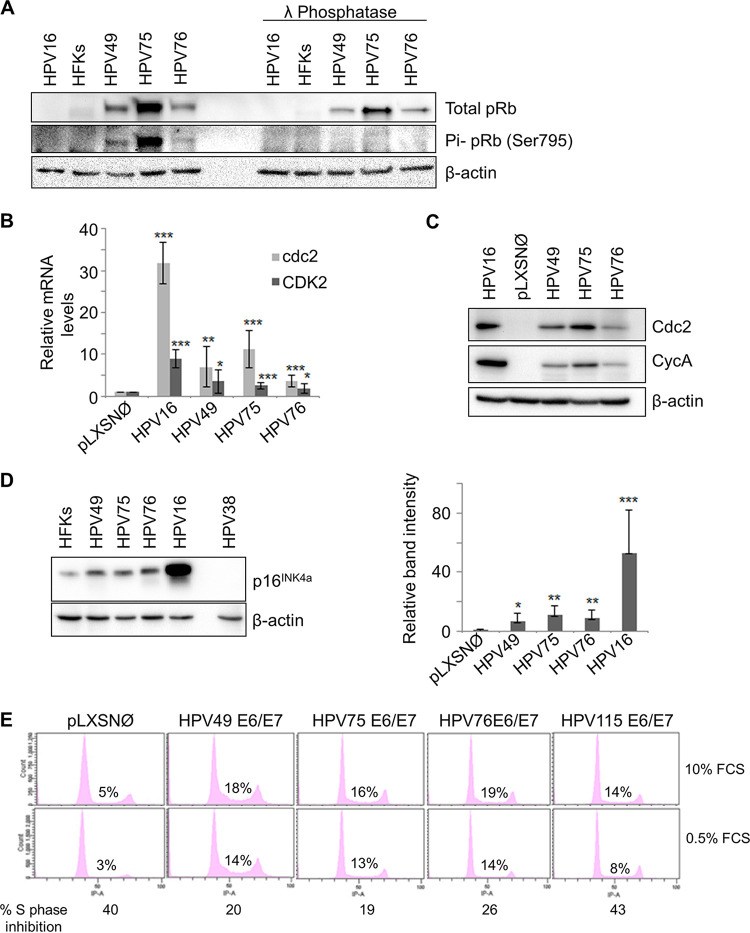
Beta-3 E6/E7 alter pRb function in cell cycle control. (A) Protein extracts from HFKs expressing or not expressing E6/E7 of the indicated HPV types were incubated with or without λPP and analyzed by IB using pRb, phosphorylated pRb (Pi-pRB) (Ser795), and β-actin antibodies. (B) Retrotranscribed total RNA was used as a template for real-time PCR with primers specific for cdc2 and CDK2 genes or GAPDH. cdc2 and CDK2 expression levels were normalized to GAPDH levels. Results shown in the histogram are the means of data from 6 independent experiments performed using HFKs from 2 independent donors. (C and D) Protein extracts from HFKs or transduced HFKs expressing E6/E7 of the different beta-3 types were analyzed by IB using the indicated antibodies (Cdc2, CycA, and β-actin [C] and p16^INK4a^ and β-actin [D, left panel]). Band intensities were quantified, normalized to β-actin levels, and then calibrated against the HFKs (D, right panel). The error bars represent the standard deviations of results from 5 independent experiments performed in HFKs from 2 independent donors (*, *P < *0.05; **, *P < *0.01; ***, *P < *0.005). (E) PHFs were transduced with empty retrovirus (pLXSN) or with retroviruses expressing E6 and E7 of the 4 beta-3 HPV types. Cell were grown in DMEM with 10% or 0.5% fetal calf serum for 48 h, and the cell cycle profile was analyzed by flow cytometry. The levels of inhibition of the S phase by serum starvation are given at the bottom of the figure.

Alteration of cellular proliferation is usually associated with deregulation of pathways controlled by the product of the retinoblastoma tumor suppressor gene (pRb). Accordingly, E7 oncoproteins from many HPV types have the ability to bind and to inactivate pRb (4). We have previously shown that HPV49 E7 inactivates pRb by promoting its hyperphosphorylation, a mechanism that differs from that of HR HPV16 E7, which leads to pRb degradation via the proteasome pathway (23). We therefore determined the status of pRb in HFKs expressing E6/E7 from HPV49, HPV75, and HPV76. HPV115 E6/E7 HFKs were not further characterized, because they rapidly entered senescence. HPV49, HPV75, and HPV76 E6/E7 HFKs have high levels of total pRb compared with mock treatment (mock) cells (HFKs) and with HPV16 E6/E7 HFKs (Fig. 2A). A specific antibody against the pRb form phosphorylated at serine 795 recognized a protein band in HPV49, HPV75, and HPV76 E6/E7 HFKs; the band was no longer visible after treatment of the total cellular extract with lambda protein phosphatase (λPP) (Fig. 2A). Thus, all three of these beta-3 HPV types are able to promote accumulation of a phosphorylated pRb form. Different levels of efficiency in promoting pRb phosphorylation were seen among the beta-3 HPV types in independent experiments, most likely reflecting small differences in the proliferative status of the different cell cultures (data not shown). However, the levels of phosphorylated pRb in beta-3 HPV E6/E7 HFKs were always considerably higher than those in untreated HFKs and HPV16 E6/E7 HFKs (data not shown). pRb phosphorylation results in a release of free and active E2F1 to E2F3, which in turn induce the expression of genes encoding positive-cell cycle regulators, such as cdc2 and cyclin A (28). Accordingly, we observed an increase of mRNA and/or protein levels of cdc2, CDK2, and cyclin A in HFKs expressing E6/E7 from HPV49, HPV75, and HPV76 compared with the mock HFKs (pLXSNØ) (Fig. 2B and C). However, the HR HPV16 appeared to be more efficient than any of the three beta-3 HPV types in increasing the mRNA and protein levels of cell cycle regulators (Fig. 2B and C).

Accumulation of the cell cycle inhibitor p16^INK4a^, an event associated with cell cycle deregulation mediated by HR HPV16 (29, 30), was detected in the HFKs expressing HPV49, HPV75, or HPV76 E6 and E7 (Fig. 2D). However, the level of p16^INK4a^ upregulation was lower in beta-3 HPV E6/E7 HFKs than in HR HPV16 E6/E7 HFKs (Fig. 2D). No p16^INK4a^ signal was detected in HPV38 E6/E7-immortalized HFKs, suggesting that HPV38 differs from the beta-3 HPVs in its cell cycle deregulation mechanisms (Fig. 2D). Next, to further compare the abilities of the different beta-3 HPV types with respect to cell cycle deregulation, we established stable lines of primary human fibroblasts (PHFs) transduced with empty retroviral vector (pLXSNØ) or carrying E6/E7 genes from all four beta-3 HPVs. Serum deprivation inhibited S-phase entry in similar manners in mock cells (pLXSNØ) and HPV115-PHFs, while PHFs expressing E6 and E7 from HPV49, HPV75, and HPV76 continued to proliferate even under serum-deprived conditions (Fig. 2E).

In summary, our data show that the pRb pathway and cell cycle control are altered in HPV beta-3 E6/E7 HFKs.

### p53 is degraded via the proteasome pathway in beta-3 HPV E6/E7 HFKs.

**FIG 3:**
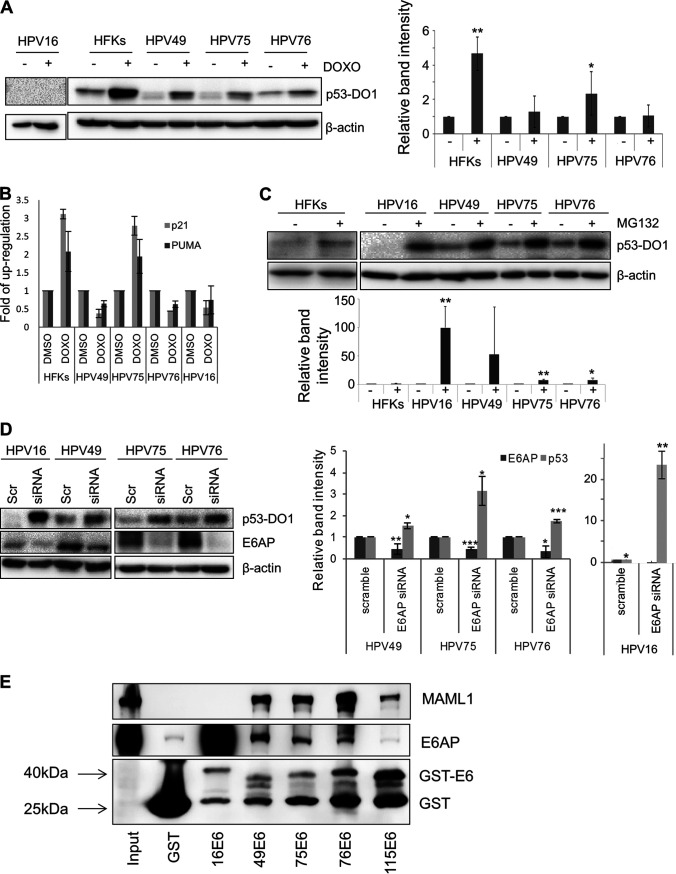
Beta-3 E6/E7 alter p53 function by a mechanism similar to that seen with HR HPV16. (A) HFKs transduced with E6/E7 from beta-3 types with HPV16 E6/E7 or nontransduced HFKs were treated for 8 h with the DNA-damaging agent doxorubicin (DOXO) used at the final concentration of 2 μg/ml or with DMSO as a control and were collected. Levels of p53 and β-actin were determined by IB using specific antibodies. Band intensities were quantified, normalized to β-actin levels, and then calibrated against the corresponding protein band levels obtained in the scramble samples (right). Results shown are the means of data from 3 independent experiments performed in primary HFKs from 2 donors (*, *P < *0.05; **, *P < *0.01). (B) Total RNA extracted from cells treated as described for panel A was retrotranscribed and used as a template for RT real-time PCR analysis of PUMA and p21 gene expression, normalized to GAPDH levels. Results shown in the histogram are the means of data from 3 independent experiments performed in one donor. (C) Nontransduced HFKs or HPV16, HPV49, HPV75, and HPV76 E6/E7-transduced HFKs were treated for 4 h with a proteasome inhibitor (MG132). Levels of p53 and β-actin were determined by IB using specific antibodies. Band intensities were quantified, normalized to β-actin levels, and then calibrated against the corresponding protein band levels obtained in the scramble samples (right). Results shown are the means of data from 2 (HFKs) independent experiments performed in primary HFKs from 2 donors (*, *P < *0.05; **, *P < *0.01). (D) HPV16, HPV49, HPV75, and HPV76 E6/E7-transduced HFKs were transiently transfected with siRNA directed against E6AP (siRNA) or with scramble (Scr). Levels of p53, E6AP, and β-actin were determined by IB using specific antibodies (left). Band intensities were quantified, normalized to β-actin levels, and then calibrated against the corresponding protein band levels obtained in the scramble samples (right). Results shown are the means of data from 2 (HPV16) or 3 independent experiments performed in primary HFKs from 2 donors (*, *P < *0.05; **, *P < *0.01; ***, *P < *0.005). (E) The indicated GST or GST/E6 fusion proteins were incubated with 1.5 mg of HNC136 total protein extract. Levels of MAML1, E6AP, and GST were determined by IB using specific antibodies.

Several studies have shown that different HPV types use different mechanisms to alter the function of the tumor suppressor p53 (4). To date, HPV49 E6 is the only beta HPV protein that is known to possess the same mechanism as HR HPV16 E6 for promoting p53 degradation via the interaction with E6AP and the proteasome pathway (23). Immunoblotting (IB) revealed that the basal levels of p53 were lower in beta-3 HPV E6/E7 HFKs than in the mock HFKs (Fig. 3A). In addition, induction of cellular stress upon doxorubicin treatment in HFKs leads to strong p53 accumulation, whereas this event is much attenuated in beta-3 HPV E6/E7 HFKs (Fig. 3A). Accordingly, although treatment with doxorubicin efficiently induced the expression of the two p53 target genes p21^WAF1^ and PUMA in HFKs, no transcriptional activation of these two genes was observed in HPV49 or HPV76 E6/E7 HFKs (Fig. 3B). However, doxorubicin treatment of HPV75 E6/E7 HFKs resulted in a considerable increase in mRNA levels of p21^WAF1^ and PUMA (Fig. 3B), suggesting that this beta HPV type is less efficient than HPV49 and HPV76 in targeting p53, which in HPV75 cells appears to retain part of its function. As expected, we did not detect p53 protein in HPV16 E6/E7 HFKs (Fig. 3A), and neither did we observe induction of expression of p53 target genes upon doxorubicin treatment (Fig. 3B).

Treatment of HPV49, HPV75, and HPV76 E6/E7 HFKs with the proteasome inhibitor MG132 led to an increase in p53 protein levels, as observed in HR HPV16 E6/E7 HFKs (Fig. 3C), indicating that E6/E7 from beta-3 HPV have the ability to target p53 for degradation. To evaluate the possible role of E6AP in beta-3 HPV-induced p53 degradation, we silenced E6AP expression with small interfering RNA (siRNA). In all beta-3 HPV HFKs, inhibition of E6AP expression led to an increase in p53 levels (Fig. 3D). It has been reported that mucosal HR HPV E6s interact with E6AP by docking upon an alpha-helical domain containing a LXXLL motif (31, 32). Other studies have also show that E6s from other HPV types, via the recognition of a LXXLL motif, bind Mastermind-like 1 (MAML1), a core component of the transcriptional activation complex that mediates the effects of the canonical Notch signaling pathway (33–36). Comparative studies of large groups of human or animal PVs showed that certain E6s preferentially bind E6AP or MAML1 (37). On the basis of these findings, we performed pulldown assays with glutathione *S*-transferase (GST)/E6 fusion proteins expressed in bacteria to evaluate the ability of beta-3 HPV E6 to interact with E6AP and MAML1, using total protein extracts from HNC136 cells as the source of MAML1 and E6AP. E6 from beta-3 HPV49, HPV75, and HPV76 formed a complex with MAML1 and E6AP (Fig. 3E). In contrast, HPV115 E6 was able to bind only MAML1. HPV16 E6 was strongly associated with E6AP in comparison to HPV49, HPV75, and HPV76 E6s, while it did not bind MAML1.

Together, these data show that beta-3 HPV49, HPV75, and HPV76 E6 proteins are able to promote p53 degradation via the interaction with E6AP and the proteasome pathway. However, HPV75 E6/E7 HFKs appear to retain p53 transcriptional activity under conditions of exposure to cellular stress.

### The transforming properties of HPV76 E6 are affected by mutations in the corresponding regions of HPV16 E6 involved in p53 and E6AP binding.

**FIG 4:**
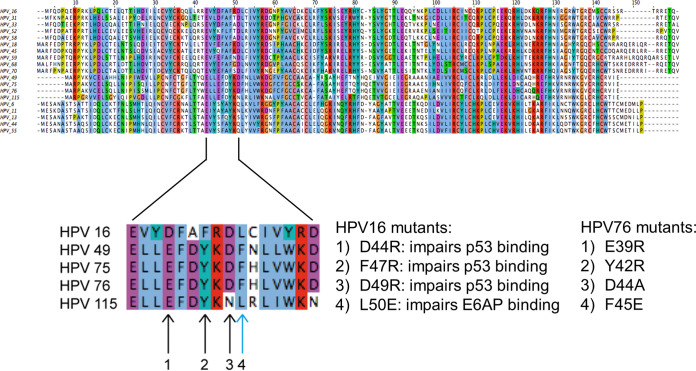
Generation of HPV76 E6 mutants. E6s from different alpha and beta HPV types were aligned. The arrows indicate the positions mutated, and the corresponding amino acid residues mutagenized in HPV76 are indicated on the right. Black arrows indicate amino acids directly involved in p53 binding, and the light blue arrow indicates the amino acid involved in binding the LxxLL motif of E6AP.

Next, we evaluated whether the ability of the beta-3 HPV E6 to target p53 is linked to their immortalization activity, using HPV76 as an example. On the basis of the information available about the structure of the E6/E6AP/p53 complex, we mutagenized 4 amino acids in HPV76 E6 (E39, Y42, D44, and F45) that correspond, respectively, to D44, F47, D49, and L50 in HPV16 E6 (Fig. 4). The negatively charged HPV16 E6 amino acids D44 and D49 are involved in the formation of a polar bridge with p53, while HPV16 E6 F47 is part of a hydrophobic core crucial for the interaction with p53 (38, 39). The amino acid HPV16 E6 L50 mediates the interaction with the LxxLL motif present in E6AP and other cellular proteins (38, 39).

**FIG 5:**
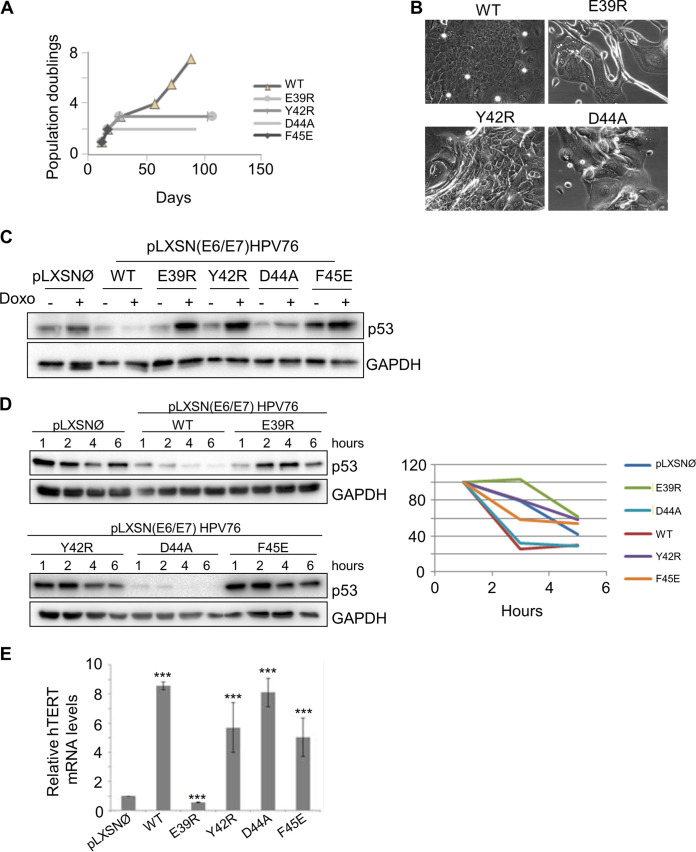
Mutation of HPV76 E6 in amino acid residues corresponding to p53 and E6AP binding sites in HPV16 E6 altered its biological activities. (A) Growth curve of primary HFKs expressing E6/E7 with WT E6 or mutated E6. PDs are reported on the vertical axis. (B) Morphology of HFKs transduced with the indicated recombinant retroviruses at PD2. The same magnification (×20) was used for all the microphotographs. (C) NIKs transduced with E7 and with WT or mutated E6, or with the empty vector (pLXSNØ) as a control, were treated with the DNA-damaging agent doxorubicin (Doxo) used for 8 h at the final concentration of 10 μg/ml. Levels of p53 and GAPDH were determined by IB using specific antibodies. (D) (Left) NIKs transduced as described in the Fig. 4D legend were treated with a protein synthesis inhibitor (cycloheximide, at the final concentration of 10 μg/ml) and collected at the indicated time points. Twenty micrograms of total protein extracts from NIKs expressing pLXSNØ, WT, and E39R E6 mutant were run on a Western blot separately from NIKs expressing Y42R, D44A, and F45E E6 mutants (upper and lower panels, respectively). Levels of p53 and GAPDH were determined by IB using specific antibodies. (Right) Graph showing quantification of p53 signal, normalized to GAPDH levels and calibrated against the levels of p53 at 2 h of treatment (set as 100%). Results shown are the means of data from 3 independent experiments. (E) Total RNA was extracted from the indicated NIKs and retrotranscribed. hTERT gene expression was determined by quantitative PCR and normalized to GAPDH. The histogram represents the quantification of the results from three independent experiments. ***, *P* < 0.005.

HFKs were transduced with a single retroviral construct carrying HPV76 E7 together with wild-type (WT) E6 or the different E6 mutants. As expected, HFKs expressing WT HPV76 E6/E7 showed an increased life span (Fig. 5A) and displayed features of highly proliferating keratinocytes (Fig. 5B). Expression of E6 mutant F45E led to rapid death of the HFKs (Fig. 5A). The HPV76 E6 E39R, Y42R, and D44A mutants were also unable to cooperate with E7 in increasing cell proliferation and subvert the senescence program, even though the cells remained in a senescent state for several days before dying (Fig. 5A and B). Next, we assessed the ability of the HPV76 E6 mutants to degrade p53 compared with WT E6. Given the very short life span of primary keratinocytes carrying the different mutated E6 open reading frames (ORFs), we generated naturally immortalized keratinocytes (NIKs) stably expressing HPV76 E7 and WT or mutated E6. Then, cells were treated with doxorubicin and analyzed for p53 levels. As expected, p53 levels increased in pLXSNØ NIKs upon doxorubicin treatment, whereas p53 was not stabilized in the doxorubicin-treated NIKs expressing HPV76 E6WT/E7 (Fig. 5C). NIKs expressing HPV76 E6 E39R, Y42R, and F45E mutants were found to have lost the ability to degrade p53, as shown by the higher p53 levels in doxorubicin-treated cells than in their untreated counterparts (Fig. 5C). In contrast, the HPV76 E6 D44A mutant showed the same ability to prevent p53 accumulation induced by doxorubicin treatment as WT E6 (Fig. 5C). To corroborate the data on the efficiency of the different HPV76 E6 mutants in targeting p53, we assessed p53 half-life by blocking *de novo* protein synthesis. NIKs expressing HPV76 E7 together with WT or mutated E6 were exposed to cycloheximide, and the p53 levels were determined at the indicated time by IB (Fig. 5D). In accordance with the data presented in Fig. 5C, the HPV76 E6 E39R, Y42R, and F45E mutants did not influence p53 half-life, in contrast to the HPV76 E6 WT and the D44A mutant, which decreased p53 half-life with similar efficiencies (Fig. 5D). Finally, we evaluated whether the HPV76 E6 mutants retained the ability to stimulate hTERT expression. Quantitative RT-PCR revealed that NIKs expressing HPV76 E6 Y42R, D44Am and F45E mutants retained a level of efficiency similar to that seen with WT E6 in upregulating hTERT expression (Fig. 5E). In contrast, no hTERT transcriptional was observed in HPV76 E6 E39R-expressing cells, indicating that this mutation affects several E6 functions or protein stability (Fig. 5E).

In summary, these results show that HPV76 E6 mutations in the corresponding regions of HPV16 E6 involved in p53 and E6AP interactions affect its transforming activity in HFKs. However, the fact that some of these mutants retain the ability seen with WT E6 to promote p53 degradation and/or activate hTERT expression indicates that these mutations must affect additional E6 properties that are essential for the extension of life span/immortalization of HFKs.

### Beta-3 HPV and HPV16 E6/E7 HFKs show some similarities in the alteration of cellular gene expression.

**FIG 6:**
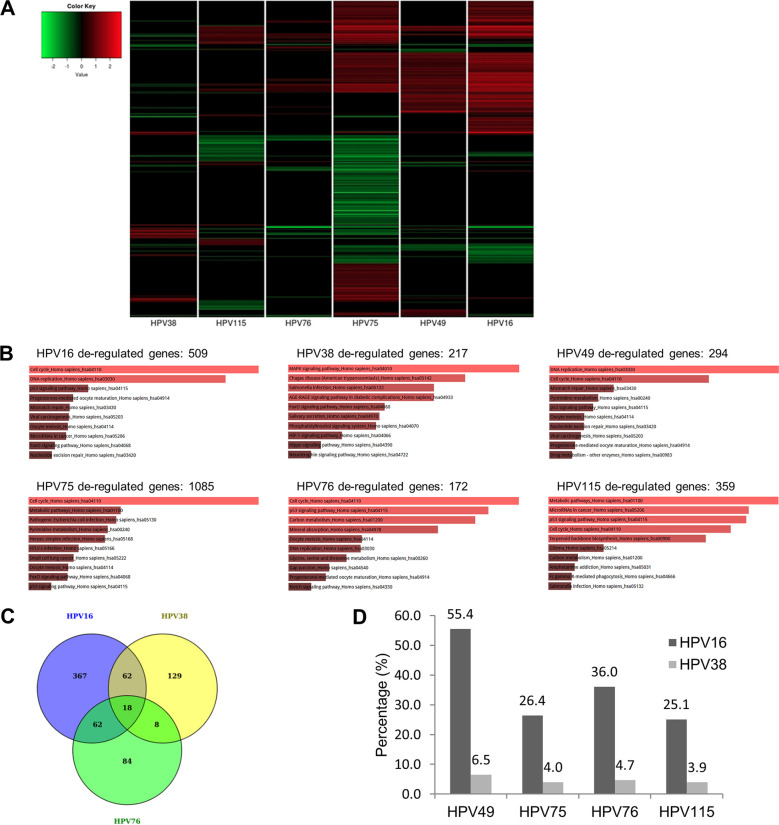
Expression of E6/E7 from the immortalizing beta-3 HPVs altered the whole expression profile of HFKs similarly to expression of HPV16 E6/E7. (A) Heat map of the significantly deregulated genes in the HFKs expressing E6/E7 from the indicated HPVs. The colors represent expression levels of the genes: red represents higher expression than that seen in empty vector HFKs, and green represents lower expression. (B) Pathway analysis (Enrichr software) of the significantly deregulated genes in cells expressing E6/E7 of the indicated HPVs. The length of the bar and the brightness of the color represent the significance of the specific pathway (combined score ranges were as follows: for HPV16, 8.25 to 72.34; for HPV38, 1.27 to 1.76; for HPV49, 6.88 to 72.91; for HPV75, 5.06 to 14.60; for HPV76, 2.57 to 6.99; for HPV115, 3.27 to 5.94). The size of each of the gene sets is indicated by the number above each histogram. AGE-RAGE, advanced glycation end product/receptor for advanced glycation end product; HIF-1, hypoxia-inducible factor 1; HTLV-1, human T-cell leukemia virus type 1; MAPK, mitogen-activated protein kinase. (C) Venn diagram of the genes deregulated in the cells expressing E6/E7 from HPV16, HPV38, and HPV76. (D) Percentages of genes of the indicated beta-3 type that are also deregulated in HPV16 or HPV38. Common genes with opposite deregulation trends have been excluded from the analysis.

To gain more insight into the biological activities of E6 and E7 from the different beta-3 HPV types, we performed RNA expression profiling of HFKs expressing E6/E7 from HPV75, HPV76, HPV115, and HPV49. We also performed RNA expression analysis of the alpha HR HPV16-E6/E7 and the beta-2 HPV38-E6/E7 HFKs, as well as of primary HFKs carrying the empty retroviral vector as a control. The genes represented in the heat map in Fig. 6 (see also Table S1 in the supplemental material) were specifically deregulated by the expression of the E6/E7 from the different HPV types, compared with the expression level of the same genes in empty vector HFKs. Of note, the hierarchical clustering analysis showed that HPV115 E6/E7-expressing HFKs, which did not show immortalization, had the most divergent expression profile from that seen with HPV16 E6/E7-expressing HFKs among the beta-3 HPV types (Fig. 6A). Pathway analysis performed by the use of Enrichr software revealed that the population of genes deregulated by all types of beta-3 HPV E6/E7 was enriched in cell cycle-related genes (Fig. 6B; see also Table S2). Because E6/E7 of the beta-3 HPV types shared similarities with E6/E7 from HPV16 in our functional assays, we assessed whether HFKs expressing E6/E7 from the beta-3 HPVs shared more deregulated genes with HPV16 or with HPV38 E6/E7 HFKs. To this end, we performed a comparative analysis, elucidated by the Venn diagram in Fig. 6C, showing the overlap in deregulated genes in HPV76-expressing HFKs compared with HPV16 and HPV38 E6/E7 HFKs (Fig. 6C). The same type of comparative analysis was done for the other beta-3 HPV types. The results, which are summarized in the histogram in Fig. 6D, show that HFKs expressing E6/E7 from beta-3 HPVs shared a higher percentage of common deregulated genes with HPV16-E6/E7 than with HPV38-E6/E7 HFKs (Fig. 6D). A separate pathway and ontology analysis of the population of genes that were found specifically deregulated both in beta-3 HPV-E6/E7 and in HPV16-E6/E7 cells confirmed their enrichment in genes related to the mitotic cell cycle, cell division, and DNA replication (data not shown). Together, these data show that E6/E7 from HPV16, HPV49, HPV75, and HPV76 shared the ability to deregulate key pathways for cell division and proliferation, which correlates well with their ability to extend the life span of keratinocytes.

10.1128/mSphere.00398-20.1 TABLE S1 List of deregulated genes in HFKs expressing E6 and E7 from the indicated HPV types in comparison to mock HFKs. Download Table S1, XLSX file, 0.5 MB. Copyright © 2020 Minoni et al. 2020 Minoni et al. This content is distributed under the terms of the Creative Commons Attribution 4.0 International license.

10.1128/mSphere.00398-20.2 TABLE S2 List of deregulated pathways in HFKs expressing E6 and E7 from the indicated HPV types in comparison to mock HFKs. Download Table S2, XLSX file, 0.01 MB. Copyright © 2020 Minoni et al. 2020 Minoni et al. This content is distributed under the terms of the Creative Commons Attribution 4.0 International license.

## DISCUSSION

Many, if not all, beta HPV types have been isolated from the skin. As a consequence, beta HPV types belonging to the 5 species (beta-1 to beta-5) have been classified, by assumption, as cutaneous HPV types. The subdivision of the different HPV types in the phylogenetic tree is based on the nucleotide sequence of the L1 gene and does not necessarily reflect their biological properties. Thus, it is plausible to hypothesize that genus beta may group HPV types with different biological properties and tissue tropisms. In line with this hypothesis, genus alpha includes mucosal low-risk and HR HPV types associated, respectively, with benign and malignant genital lesions as well as benign cutaneous HPV types responsible for the development of common skin warts. Many previous epidemiological studies determined the prevalence of beta HPV types at different anatomical sites and provided evidence for their tropism for mucosal and cutaneous tissues (15–20). Regarding the biological properties, only a limited number of beta HPV types have been studied so far (reviewed in reference 4). Several beta HPV types have developed different mechanisms involved in altering the functions of the p53 tumor suppressor. For instance, beta-2 HPV38 E7 promotes the accumulation of the p53 antagonist ΔNp73α (24, 25), whereas beta-2 HPV23 E6 blocks the UV-induced p53 activation by interacting directly with HIPK2 (homeodomain-interacting protein kinase 2), a cellular kinase responsible for p53 phosphorylation at serine 46 upon UV irradiation (40). Interestingly, beta-3 HPV49 E6, similarly to mucosal HR HPV16 E6, binds and promotes degradation of p53 via the proteasome pathway (23). In addition, E6 and E7 from HPV16 and HPV49 immortalize primary HFKs with similar levels of efficiency (23). Here, we also show that E6/E7 from HPV75 and HPV76 were able to subvert the senescence program of the HFKs and promoted an increase in life span as well as immortalization. In contrast, HPV115 E6/E7 HFKs rapidly entered senescence, as observed for primary cells. Our findings also show that, similarly to HPV49 E6, HPV75 and HPV76 E6s are able to promote p53 degradation, which appears to be in part mediated by the ubiquitin ligase enzyme E6AP. The fact that HPV115 E6 and E7 do not immortalize primary HFKs could be attributed to the inability of E6 to target p53. On the basis of the HPV16 E6/E6APpep/p53 structure, the HPV16 E6 region spanning residues D44 to D49 (equivalent to E39 to D44 in HPV76 E6) provides 3 key intermolecular contacts (two salt bridges and a stacking interaction) with p53 that are mediated by D44, F47 and D49 E6 residues (38). All 3 amino acids are conserved in E6 from HPV49, HPV75, and HPV76, whereas only 2 of these residues are conserved in HPV115 E6. Indeed, HPV16 E6 D49 corresponds to an asparagine in HPV115. The D→N substitution would prevent the formation of a salt bridge with H115 of p53. On the basis of the well-characterized three-dimensional structure of HPV16 E6 (38, 39), we mutagenized E6 of HPV76 in four conserved amino acids essential for the formation of an E6/E6AP/p53 ternary complex, namely, E39R, Y42R, D44A, and F45E. All four mutants were unable to cooperate with the corresponding E7 in the HFK immortalization assay. In addition, HPV76 E6 E39R, Y42R, and F45E lost the ability to induce p53 degradation. Our set of experiments using HPV76 E6 mutants also revealed that the ability to degrade p53 does not appear to be sufficient for the extension of the life span of primary HFKs. In fact, although the HPV76 D44A E6 mutant retained the ability of WT E6 to induce p53 degradation, it did not cooperate with E7 in the extension of the life span of HFKs, indicating that the D44A mutation alters additional and still unidentified crucial functions of the viral protein. In addition, three HPV76 E6 mutants (Y42R, D44A, and F45E) activated hTERT expression with efficiency similar to that seen with the HPV76 E6 wild type, providing evidence that they retain some functionality. Taken together, our data show that HPV types from the beta-3 genus alter p53 function by inducing its degradation with a mechanism similar to that used by the mucosal HR HPV types. However, our findings do not exclude the possibility that E6 and E7 from beta-3 HPV may also use alternative and still-uncharacterized mechanisms to block the function of p53 to safeguard cellular DNA integrity and allow the propagation of cells with DNA damage, including another ubiquitin ligase enzyme. White and colleagues, in agreement with our findings, showed that beta-3 HPV76 E6 expression in human keratinocytes reduced p53 levels; however, they did not observe any interaction with E6AP (41, 42). In the same study, it was also shown that beta-3 HPV76 E6 binds the transcriptional coactivator mastermind-like 1 (MAML1), similarly to others beta HPV E6 proteins (43). Another recent study analyzed the interactions of many HPV E6 proteins with their main cellular targets, i.e., E6AP and MAML1 (37). The findings provided evidence that the different E6 proteins from alpha or beta HPV types have the ability to perform mutually exclusive binding to either E6AP or MAML1 (37). In this study, we showed that beta-3 HPV E6s can bind both cellular proteins, although the E6/E6AP interaction appears to be weaker than the E6/MAML1 interaction. In support of our data, another study also documented the weak binding of beta-2 HPV38 E6 with E6AP (44).

With regard to the ability of E6 and E7 to drive the cells through the cell cycle and guarantee their unscheduled proliferation, mucosal HR and beta HPVs appear to use different mechanisms. Whereas E7 from mucosal HR HPV types induces degradation of the hypophosphorylated form of pRb, an event that leads to release of E2F and activation of its target genes, all beta HPVs studied to date, including the beta-3 types, induce pRb hyperphosphorylation (23). We might hypothesize that this difference in the mechanism of beta HPV types can be explained by the fact that the presence of beta HPV proteins leads to activation of the kinase complexes involved in pRb phosphorylation. Because only the hypophosphorylated form of pRb can interact with E7, the hyperphosphorylation of pRb promoted by beta HPV types was able to prevent its association with E7 and its possible degradation. However, beta-3 HPV types appear to be less efficient than HR HPV in inactivating pRb, as shown by the lower level of induction of E2F1 target genes in HFKs expressing E6 and E7 from beta-3 HPVs than in HPV16 E6/E7-expressing HFKs. Previous studies have shown that expression of HPV16 E7 is associated with an increase in intracellular levels of p16^INK4a^ (29, 30). Our data showing that p16^INK4a^ is induced in beta-3 HPV E6/E7 HFKs, but not in beta-2 HPV38 E6/E7 HFKs, further underline the functional similarity between beta-3 and mucosal HR HPV types.

Finally, analysis of the expression profiles revealed that HFKs stably expressing E6/E7 oncogenes from beta HPV49, HPV75, and HPV76 share some similarities with HPV16 E6/E7 HFKs. In addition, the expression profiles of HPV75, HPV76, and HPV49 E6/E7 HFKs diverged substantially from those of HFKs expressing beta-3 HPV115 E6/E7 as well as from those of the beta-2 HPV38 E6/E7 HFKs.

In conclusion, the biological findings support the concept that beta-3 HPV types share several properties with mucosal HR HPV types, which could be explained by their ability to infect mucosal epithelia. In support of this hypothesis, two independent studies revealed that beta-3 HPV types are more frequently detected in the mucosal epithelia than the beta-1 and beta-2 HPV types, which are abundantly present in keratinized tissues (17, 22). However, no studies have reported the detection of beta-3 HPV DNA in human cancers. We recently provided evidence that expression of beta-2 HPV38 E6 and E7 genes in the skin of Tg animals promotes the accumulation of a large number of UV-induced DNA mutations and, consequently, sSCC development. Importantly, deletion of the viral oncogenes after formation of UV-induced skin lesions did not affect the tumor growth, supporting the concept that viral proteins act only at an initial stage of carcinogenesis, by potentiating the deleterious effects of UV radiation (27). Experiments with K14 HPV Tg animals showed that beta-3 HPV49 E6 and E7, similarly to HPV16 E6 and E7, strongly increased the cancer susceptibility in the upper digestive tract upon exposure to 4NQO and accumulation of tobacco-induced DNA mutations (26). In these animals, cancer development tightly correlated with accumulation of a large number of DNA mutations, which correspond to the tobacco-induced mutational signature (27). Thus, on the basis of the findings from the experimental models employed *in vivo* as described above, we can speculate that the presence of beta-3 HPVs in humans might increase their susceptibility to other risk factors but that the beta-3 HPVs could be lost after development of malignant lesions. Future studies are required to determine the prevalence of beta-3 HPV types in premalignant and malignant lesions at different anatomical sites to evaluate their possible role in carcinogenesis. A few previous studies highlighted a possible synergism of beta HPV types and UV in conjunctival cancer, as observed in skin carcinogenesis (45, 46). Interestingly, we recently observed that the prevalence of beta-3 HPV 75 is higher in conjunctival healthy mucosa and lesions than in other epithelia (47). Future molecular and epidemiological studies are warranted to further confirm the mucosal tropism of beta-3 HPV types and their possible link to human diseases.

## MATERIALS AND METHODS

### Cell culture and treatments.

NIH 3T3, HNC136, and primary human neonatal foreskin fibroblasts (PHFs) were cultivated in high-glucose (4.5 g/liter) Dulbecco’s modified Eagle’s medium (DMEM) supplemented with 10% fetal calf serum (FCS). J2 cells were cultivated in high-glucose (4.5 g/liter) DMEM supplemented with 10% bovine serum. HFKs were isolated from healthy patients and cocultured with NIH 3T3 feeder layers in FAD medium containing 3 parts Ham’s F12 medium, 1 part DMEM, 5% FCS, insulin (5 μg/ml), epidermal growth factor (10 ng/ml), cholera toxin (8.4 ng/ml), adenine (24 μg/ml), and hydrocortisone (0.4 μg/ml). NIKs were cocultured with J2 feeder layers in FAD medium. Feeder layers were prepared by mitomycin treatment (0.5 mg/ml) for 2 h. For the determination of PDs, cells were cultured in 25-cm^3^ flasks and trypsinized when they reached approximately 80% confluence. PDs were calculated by taking into consideration the number of passages and the split ratio.

For the doxorubicin treatment, 3.5 × 10^5^ HFKs or HFKs expressing E6/E7 of the different HPVs were seeded in 6-well plates. After 24 h, cells were treated with doxorubicin (Sigma-Aldrich D1515, 2 μg/ml) or dimethyl sulfoxide (DMSO) and collected at 8 h.

For MG132 treatment, 3.5 × 10^5^ HFKs or HFKs expressing E6/E7 of the different HPVs were seeded in 6-well plates. After 24 h, cells were treated with MG132 (Sigma-Aldrich C2211, 10 μM) or DMSO and collected at 4 h.

For cycloheximide treatment, 3.5 × 10^5^ NIKs expressing E6/E7 of the different HPVs or pLXSNØ (as a control) were seeded in 6-well plates. After 24 h, cells were treated with cycloheximide (Ozyme 2112S, 10 μg/ml) or DMSO and collected at 1, 2, 4, and 6 h.

For λPP treatment, 20 μg of total protein extracts was treated with λPP for 30 min at 30°C.

For fluorescence-activated cell sorter (FACS) staining, cells were grown in DMEM with 10% or 0.5% fetal calf serum for 48 h. The cells were then collected, washed twice in phosphate-buffered saline (PBS), and fixed in 70% ethanol for 30 min in ice. Cells were pelleted and washed with PBS 3 times. Cells were resuspended in 500 μl of PBS with 20 μg/ml propidium iodide and 10 μg/ml RNase A and incubated at room temperature for 30 min. Analysis was performed using a FACSCanto flow cytometer (Becton, Dickinson).

### Retroviral infections.

The E6/E7 ORFs from the HPV16, HPV49, HPV75, and HPV76 WT and mutant strains and HPV115 were cloned by PCR in pLXSN retroviral vector (Clontech, Le Pont-de-Claix, France). Retroviral supernatants were generated by transient transfection of Phoenix cells (amphotropic virus) and used to infect HFKs and PHFs as previously described (17). After infection, the HFKs and PHFs were selected with G418 (100 μg/ml) for 7 days.

### Immunoblot analysis and antibodies.

Total protein extraction, sodium dodecyl sulfate (SDS)-polyacrylamide gel electrophoresis (PAGE), and IB were conducted as described previously. The following antibodies were used: β-actin (C4; MP Biomedicals), phospho-pRb Ser795 (Cell Signaling catalog no. 9301), total pRb (BD Pharma catalog no. 554136), Cdc2 (ab-2) (Calbiochem catalog no. CC01), cyclin A (H-432) (Santa Cruz catalog no. sc751), p16^INK4a^ (catalog no. DCS-50; NovoCastra), p53 DO1 (Santa Cruz catalog no. sc126), E6AP-330 (Sigma catalog no. E8655), MAML1 (Cell Signaling catalog no. 12166), GST (Cell Signaling catalog no. 2622S), and GAPDH (glyceraldehyde-3-phosphate dehydrogenase) (6C5) (Santa Cruz catalog no. sc-32233). Images were taken using a ChemiDoc XRS imaging system (Bio-Rad).

### RT-PCR and qPCR analysis.

Total RNA was extracted from mammalian cells with a NucleoSpin RNA kit (Macherey-Nagel). cDNA was synthetized with a RevertAid RT reverse transcription kit (Thermo Scientific) using random primers. Quantitative PCR (qPCR) was performed using Mesa Green qPCR Master Mix Plus for SYBR assay (Eurogentec) with the primers listed in Table 2.

**TABLE 2:** qPCR primer sequences

Gene	Primer sequence
cdc2	F: 5′-AATCTATGATCCAGCCAAACGAA-3′
	R: 5′-TTCTTAATCTGATTGTCCAAATCATTAAA-3′

CDK2	F: 5′-GGCTGCATCTTTGCTGAAAT-3′
	R: 5′-CCCAGAGTCCGAAAGATCCG-3′

p21	F: 5′-GACACCACTGGAGGGTGACT-3′
	R: 5′-CCACATGGTCTTCCTCTGCT-3′

PUMA	F: 5′-GGATGAAATTTGGCATGGGGTCT-3′
	R: 5′-GGACAAGTCAGGACTTGCAG-3′

GAPDH	F: 5′-AAGGTGGTGAAGCAGGCGT-3′
	R: 5′-GAGGAGTGGGTGTCGCTGTT-3′

hTERT	F: 5′-TTCAAGGCTGGGAGGAACAT-3′
	R: 5′-ACATGCGTGAAACCTGTACG-3′

### Gene silencing.

Gene silencing of E6AP was achieved using synthetic siRNA. siRNAs (Dharmacon L-005137-00-0005) or scrambled RNAs (Eurofins) were transfected at a concentration of 10 nM using Lipofectamine 2000 according to the standard protocol (Invitrogen).

### GST pulldown.

pGEX/E6 plasmids were prepared by PCR cloning, and the E6 ORFs were cloned at the GST C terminus. pGEX/E6 expression plasmids were transformed into Escherichia coli BL21 bacteria (Rosetta), and GST/E6 fusion proteins were induced by the use of isopropyl β-d-1-thiogalactopyranoside (IPTG). GST/E6 proteins were extracted from 50 ml E. coli BL21 pellet by sonication in PBS–1% Triton X-100 buffer and bound to glutathione Sepharose 4B beads (Dutscher, catalog no. 17075601) following the manufacturer’s instructions. HNC136 total cell extracts were precleared for 3 h at 4°C, and approximately 1.5 mg of the precleared extract was incubated with GST-E6 or GST beads. Samples were washed using the protein extraction buffer (20 mM Tris [pH 8], 200 mM NaCl, 1 mM EDTA, 1% NP-40, 2 mM dithiothreitol [DTT], 0.1 mM Na_3_VO_4_, 10 mM NaF, and protease inhibitor cocktail [Roche]). The beads were suspended in Laemmli buffer, and the eluted proteins were run on an SDS-PAGE gel.

### Microarray-based whole-genome expression profiling and data analysis.

Similar PDs were used for the different HFKs, and the RNA profiling assays were performed in duplicate or triplicate as follows: (i) for pLXSN HFKs, 7.5 PDs (three samples); (ii) for HPV16 HFKs, 11.5 PDs (two samples); (iii) for HPV49 HFKs, 10.5 PDs (two samples); (iv) for HPV75 HFKs, 10.5 PDs (three samples); (v) for HPV76 HFKs, 11 PDs (three samples); for HPV115 HFKs, 5 PDs (three samples); for HPV38 HFKs, 15.5 PDs (two samples). Genome-wide gene expression profiling analysis was performed by the use of Illumina HumanHT-12 v4 Expression BeadChips (24,000 annotated genes covered). Candidate probe sequences included on the HumanHT-12 v4 Expression BeadChip derive from the NCBI RefSeq (Build 36.2, Rel22) and the UniGene (Build 199) databases. Using an Illumina TotalPrep RNA amplification kit (Ambion), 500-ng volumes of extracted RNAs were converted to cDNAs and subsequently to biotin-labeled single-stranded cRNAs. The distribution of homogeneous *in vitro* transcription products (cRNAs) was checked with an Agilent 2100 Bioanalyzer instrument and an RNA 6000 Nano kit. A 750-ng volume of biotin-labeled cRNAs of the 7 samples (including 2 controls) was hybridized overnight to 4 HumanHT-12 Expression BeadChips. Subsequent steps included washing, streptavadin-Cy3 staining, and scanning of the arrays on an Illumina BeadArray reader. Illumina Genome Studio software v2010.2 was used to obtain the signal values (AVG-Signal), with no normalization and no background subtraction. Data quality controls were performed using internal controls present on the HumanHT-12 BeadChip and were visualized as a control summary plot and for each sample as noise-to-signal ratios calculated by P95/P05 signal intensities. All samples had P95/P05 ratio values of >10, defined as the sample quality threshold (data not shown).

Differential expression analysis was performed using BRB-ArrayTools software v4.2 (48). The raw signal intensities of all samples were subjected to log transformation and quantile normalization without background subtraction, with the exclusion of any probe showing excess dispersion (defined as >85% of individual probe values differing from the median by more than 1.5-fold). Class comparison for microarray analysis was performed using the *t* test method to identify differentially expressed probes. Probes with a *P* value of <0.001, a minimum fold change value of 1.5, and a false-discovery rate of <0.01 were considered to be significantly differentially expressed. The different conditions were then hierarchically clustered (complete-linkage clustering) based on the Pearson distance of the log_2_ fold change value for each gene.

### Statistical analysis.

Statistical significance was determined using Student's *t* test. The levels of statistical significance for each experiment (*, *P < *0.05; **, *P < *0.01; ***, *P < *0.005 [or not significant]) are indicated in the corresponding figures. The error bars in the graphs represent the standard deviations.

### Data availability.

The microarray experiments were performed in a MIAME-compliant manner, and the resulting data have been deposited in the NCBI Gene Expression Omnibus (GEO) database under accession number GSE100681.
